# Kirenol Inhibits the Function and Inflammation of Fibroblast-like Synoviocytes in Rheumatoid Arthritis *in vitro* and *in vivo*

**DOI:** 10.3389/fimmu.2019.01304

**Published:** 2019-06-06

**Authors:** Jing Wu, Qiang Li, Li Jin, Yuan Qu, Bi-Bo Liang, Xiao-Tong Zhu, Hong-Yan Du, Li-Gang Jie, Qing-Hong Yu

**Affiliations:** ^1^Rheumatology and Clinical Immunology, ZhuJiang Hospital,Southern Medical University, Guangzhou, China; ^2^School of Laboratory Medicine and Biotechnology, Southern Medical University, Guangzhou, China

**Keywords:** kirenol, Rheumatoid arthritis, fibroblast-like synoviocytes, IL-6, migration, invasion

## Abstract

Kirenol is a diterpenoid extracted from the Chinese herbal medicine *Siegesbeckiae*. *Siegesbeckiae* has been used to treat Rheumatoid arthritis (RA) in China for several centuries. RA is characterized by the proliferation of synoviocytes in inflamed synovia, as well as by their expression of inflammatory cytokines. In the present study, we found that Kirenol inhibited the migration, invasion, and proinflammatory of IL-6 secretion of RA-associated synovial fibroblasts (FLS) at a concentration of 100–200 μg/ml *in vitro*. Proinflammatory cytokines production and synovium hyperplasia and cartilage erosion were also inhibited in a collagen-induced arthritis (CIA) mouse model upon Kirenol treatment. Together, our results thus confirm that Kirenol has potent therapeutic efficacy in RA owing to its ability to suppress negative FLS activities.

## Introduction

RA is a chronic and refractory autoimmune joint disease characterized by the proliferation of synoviocytes in the inflamed synovia, and by the expression of inflammatory cytokines on these cells (1). Synovial fibroblasts (FLS) promote joint destruction via their attachment to the cartilage, and thus are key mediators of the pathogenesis of RA (2). Although there are many RA treatment options available, including traditional disease-modifying antirheumatic drugs (DMARDs) as well new and effective biologicals agents, these treatments ultimately induce remission in only 20–68% of patients (3). Moreover, there is still ample opportunity for the development of novel drugs capable of inhibiting synovial hyperplasia. Kirenol is a diterpenoid compound derived from *Herba Siegesbeckia* that has been traditionally used in China to treat RA for centuries. Kirenol has been suggested to exhibit primary anti-inflammatory and anti-rheumatic activities (4, 5). The active ingredient Kirenol in *Herba Siegesbeckia* extracts was shown to reduce the inflammatory pathology in collagen induced arthritis (CIA) model rats, and additional studies suggest that Kirenol is able to suppress the production of IL-1β and TNF-α in the serum of adjuvant arthritis model animals (5, 6). While multiple studies have thus demonstrated the anti-inflammatory properties of Kirenol, there is still very limited information available regarding the specific mechanisms and dynamics whereby Kirenol affects RA-associated FLS cells both *in vitro* and *in vivo*. The inflammatory milieu in the synovial compartment is regulated by a complex cytokine network. Many pro-inflammatory cytokines such as TNF-α, IL-1β, and IL-6, are thought to contribute to the pathological development and progression of RA (7). Activated FLS cells secrete large quantities of IL-6 and IL-8 (8). Antibodies directed against TNF-α and IL-6 have shown efficacy for the treatment of RA, consistent with the fact that joint destruction is positively correlated with pro-inflammatory cytokine levels in the serum or synovial tissue. Multiple cytokines and matrix metalloproteinases (MMPs) are present in the synovium of RA patients, where they play important roles in the maintenance of inflammatory responses (9, 10). Certain proteins and cytokines, including IL-6, IL-8, TNF-α, IL-1β, and MMP-1, 2, 3, 9 have been identified as diagnostic indicators of RA and as possible therapeutic targets. As such, any effort to determine how Kirenol affects FLS cells necessitates an investigation of its effects on cytokine production.

The pathophysiology of RA involves chronic inflammation and pannus formation in the synovial membrane, which can lead to the destruction of articular cartilage and bone. As these pro-inflammatory cytokines and MMPs are specifically involved in the pathogenesis in RA and are highly expressed in the serum and synovial fluid of RA patients, we hypothesized that these factors may be downregulated by Kirenol. The aims of this study were therefore to evaluate whether Kirenol treatment leads to decreased production of these factors by RA-associated FLS cells, and to explore the underlying molecular mechanisms governing such regulatory activity.

## Materials and Methods

### Human FLS Culture

Synovial tissue samples were obtained from the knees of five patients with active RA (as diagnosed according to the 2010 Rheumatoid arthritis classification criteria) during knee joint arthroscopic operations. The synovial tissue was cut into 1–2 mm^3^ pieces and distributed evenly in a culture flask. After 4 h, this flask was inverted and the synovial tissue was cultured in DMEM containing 10% fetal calf serum, 100 U/ml penicillin, and 100 μg/ml streptomycin in a humid incubator containing 5% CO_2_. Cell media was changed every 3–4 days. The FLS cells were grown in a monolayer, and cells between the third and sixth generations were used for all experiments.

### Cell Viability Assays

Cell viability was detected using the CCK-8 kit (Dojindo, China) according to the provided instructions. Briefly, cultured RA-FLS cells were plated in 96-well plates at a density of 1 × 10^3^ cells/well in DMEM containing 10% FBS. Cells were then incubated with Kirenol (50, 100, and 200 μg/ml; Herbpurity, China) for another 24 h. Human IL-17A (100 ng/ml, R&D, USA) and TNF-α (100 ng/ml, R&D, USA) were used as positive controls. After this incubation period, 10 μL of the CCK-8 solution was added to each well and cells were incubated for 4 h. The absorbance at 450 nm was then measured via a microplate reader.

### Quantitative PCR

RA-FLS cells were seeded in 24-well plates at a density of 2 × 10^4^ cells/well for 24 h, and were then treated with Kirenol at concentrations of 50, 100, or 200 μg/ml for 4 h, with positive controls employed as above. Total FLS RNA was then isolated at appropriates using the Trizol reagent (Invitrogen, USA) according to the manufacturer's protocols. Reverse transcription was conducted using a first-strand cDNA synthesis kit (TaKaRa, China). To assess IL-6, IL-8, MMP1, MMP2, MMP3, MMP9, NFκB P50, NFκB P65, MAPK, JNK, and JAK expression, real-time PCR was performed using a SYBR Premix ExTaq kit (TaKaRa, China). Resultant heatmap figures were prepared using the R software [package(heatmap)].

### ELISAs

After being treated as described above, 2 × 10^4^ RA-FLS cells were treated with Kirenol for 4 or 24 h. Supernatants were then collected to measure IL-6, IL-8, IL-1β, and TNF-α. For *in vivo* experiments, murine serum was similarly used for cytokine detection. ELISA kits used included those specific for IL-1β (R&D, USA), TNF-α (Thermo Fisher Scientific, USA), IL-6 (Thermo Fisher Scientific, USA), IL-8 (Thermo Fisher Scientific, USA). The optical density (OD) value for each sample was determined at 450 nm.

### Western Blotting

RIPA lysis buffer (50 mM Tris-Cl pH 7.4, 150 mM NaCl, 1% Triton X-100, 1% sodium deoxycholate, 0.1% SDS), containing protease and phosphatase inhibitors as well as phenylmethanesulfonyl fluoride (PMSF), was used to lyse and collect protein from cell samples. Protein was then loaded onto 8% polyacrylamide Tris/glycine gels and separated at 80 V for 30 min, followed by 110 V for 1 h, and samples were then transferred to a nitrocellulose membrane at 100 V for 2 h. After blocking, the membranes were probed using the MAPK Family Antibody Sampler Kit (Cell Signaling Technology, USA), NF-κB Pathway Sampler Kit (Cell Signaling Technology, USA), or Phospho-Jak Family Antibody Sampler Kit (Cell Signaling Technology, USA). Phospho-antibodies were diluted to 1:100, while all others were diluted to 1:500. After chemiluminescence development (SignalFire™ ECL Reagent, Cell Signaling Technology, USA), gel images were scanned and analyzed using the Image J (v1.52) image processing software.

Murine synovial tissues were taken from around the hip joints, as described in our previously research method (11). For murine synovium samples, western blotting was performed as above, using IL-6, IL-8, and TNF-α antibodies purchased from Biomathematics and Statistics Scotland (China).

### Measures of FLS Migration and Invasion

#### Wound Healing Assay

To demonstrate the effects of Kirenol on the migratory capacity of FLS, a wound healing assay was performed. 2 × 10^5^/well RA-FLS were seeded in 24-well plates for 24 h, after which a 200 μl pipette tip was used to create a straight scratch wound in the monolayer. Cells were then incubated with Kirenol (50, 100, or 200 μg/ml) for an additional 48 h, with cell being imaged after 0, 4, 24, and 48 h. Image J (v1.52) was used to analyze the migratory wound healing dynamics.

#### Transwell Migration and Invasion Assays

To further explore the effects of Kirenol on cellular responses, chemotaxis assays were performed using transwell chambers with an 8.0 μm pore size (Corning, USA). Cells were incubated with Kirenol concentrations as described above for 24 h, and then a total of 2 × 10^5^ FLS in serum-free DMEM were added to the upper chambers of these Transwell systems for 8 h. In addition, 600 μl of DMEM medium containing 10% FBS was added to the lower chamber of each well in a 24-well plate. In parallel, similar invasion assays were performed using an 8.0 μm PET membrane (Corning, USA). For this experiment, FLS were seeded at a density of 1 × 10^5^/ml and were grown in DMEM for 12 h. Cells that failed to migrate were removed with a cotton swab, after which the membranes were fixed with 4% paraformaldehyde for 30 min and then stained with 0.1% crystal violet. Migration was quantified by counting the number of stained cells that had migrated to the lower side of the filter using an optical microscope. The average of the number of invading cells from the six random fields of view after normalization to control were used to determine rates of chemotaxis/invasion.

### Murine Experiments

#### Arthritis Model Development

CIA was induced in 9-week-old male DBA/1 mice. Mice were purchased from HuFukang Biotechnology Co., China (license number: SCXK (Jing) 2014-0004). All experiments were performed in accordance with the guidelines of the local animal ethics committee. A total of 15 mice were divided into 3 groups, and received 0, 7.5, or 30 mg/kg Kirenol q.d. Mice were treated with Kirenol for 1 week before being immunized with 100 μg of bovine type II collagen and complete Freund's adjuvant (CFA; 1:1, Xinbosheng, China and Sigma, Japan) by injection at the tail base. A booster injection was administered on day 21, at which time a total of 100 μg collagen II was administered in Freund's incomplete adjuvant (Sigma, Japan). Assessment of arthritis in each limb of these arthritic model mice was then performed via visual scoring from 0 to 4. A maximal score for an individual animal was 16 (12). The weight of each mouse was also recorded once per week.

#### Histological Scoring

Joints were removed from CIA model mice and fixed in 10% formalin, after which they were decalcified in 10% EDTA, embedded in paraffin, and stained with hematoxylin and eosin (H&E) for light microscopy. Infiltration of inflammatory cells, transformation of the synovial lining, cartilage destruction, and pannus formation were independently scored in a blinded manner from 0 to 3 as in previous studies (13). Of the four limbs analyzed per animal, the maximum score for each category was used, with a maximum possible histological score of 12. Synovial inflammation and bone erosion scores were also performed as described previously, with a maximum possible score of 4 (14). Synovial inflammation was scored as follows: 0- no inflammation; 1- slight synovitis with some cell infiltration; 2- moderate synovitis with moderate cell infiltration; 3- extensive synovitis with a moderate number of infiltrating cells; 4- extensive and severe synovitis, with the presence of numerous inflammatory cells. Bone erosion was scored as follows: 0- no erosion; 1- small areas of resorption; 2- numerous areas of resorption; 3- extensive osteolysis; 4- extensive and severe osteolysis.

#### Immunohistochemistry

Joints sections were deparaffinized and washed with Tris-buffered saline (TBS) for 10 min and distilled water for 10 min, after which antigen retrieval was performed via heating samples in citrate buffer for 15 min. Samples were then incubated with primary antibodies against IL-6 (Servicebio, USA), IL-8 (Servicebio, USA), and TNF-α (Servicebio, USA), (1:50) at 4°C for 12 h, followed by incubation with a secondary antibody (goat anti rabbit, 1:50, servicebio, USA) at room temperature for 50 min. For antigen visualization, DAB solution was used for color development for 5 min, after which the Image J (v1.52) and IHC Toolbox.jar (USA) programs were used for image analysis.

### Statistical Analysis

Data are presented as means ± standard deviation. Differences among groups were analyzed via the Kruskal-Wallis test (more than two groups) or Mann-Whitney *U*-test (two groups) using GraphPad Prism v5.0 (USA). Differences were considered to be statistically significant at *p* < 0.05.

## Results

### Kirenol Inhibits RA-FLS Proliferation

**Figure 1:**
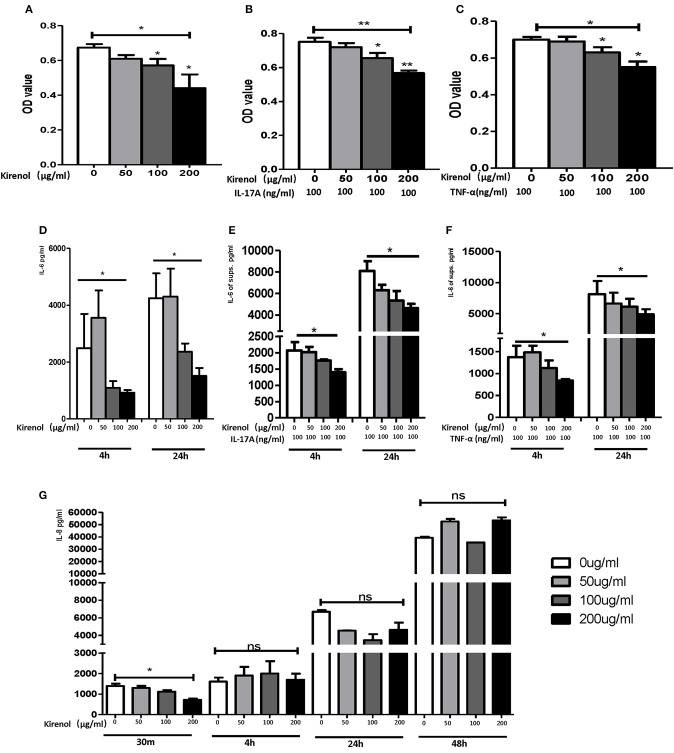
**(A)** Kirenol inhibited FLS in a dose-dependent manner; **(B)** Kirenol inhibited the proliferation of FLS stimulated with IL-17A (100 ng/ml); **(C)** Kirenol inhibited the proliferation of FLS stimulated with TNF-α (100 ng/ml); **(D)** Kirenol inhibited the secretion of IL-6 by FLS in a dose-dependent manner after 4 and 24 h. **(E)** Kirenol inhibited the secretion of IL-6 by FLS stimulated with IL-17A in a dose-dependent after 4 and 24 h. **(F)** Kirenol inhibited the secretion of IL-6 by FLS stimulated with TNF-α in a dose-dependent after 4 and 24 h. **(G)** Kirenol inhibited the secretion of IL-8 by FLS in a dose-dependent manner after 30 min but not at other time points. *p < 0.05, **p < 0.01 as assessed by the Kruskal-Wallis test and the Mann-Whitney *U*-test.

To determine whether Kirenol affects the proliferation of FLS, cells were stimulated with Kirenol (50, 100, or 200 μg/ml) and control in medium containing DMEM. As shown in Figure 1A, Kirenol impaired the proliferation of FLS in a dose-dependent manner. Even when FLS were stimulated with inflammatory cytokines as a positive control (TNF-α 100 ng/ml and IL-17A 100 ng/ml), Kirenol was still able to mildly inhibit their proliferation (Figures 1B,C).

### Kirenol Inhibits the Secretion of Cytokines by RA-FLS

As shown in Figures 1D–F, Kirenol inhibited the secretion of IL-6 by FLS with a dose-dependent manner even when cells were stimulated using TNF-α and IL-17A. However, Kirenol only significantly inhibited FLS IL-8 secretion after 30 min (Figure 1G), and no changes in IL-8 secretion were observed at any time in the TNF-α and IL-17A-stimulated groups. We were not able to detect significant levels of IL-1β or TNF-α in FLS supernatants at any time.

### Kirenol Downregulates IL-6, IL-8, MMP-9, MAPK, P65, P50, and JAK Expression in RA-FLS

**Figure 2:**
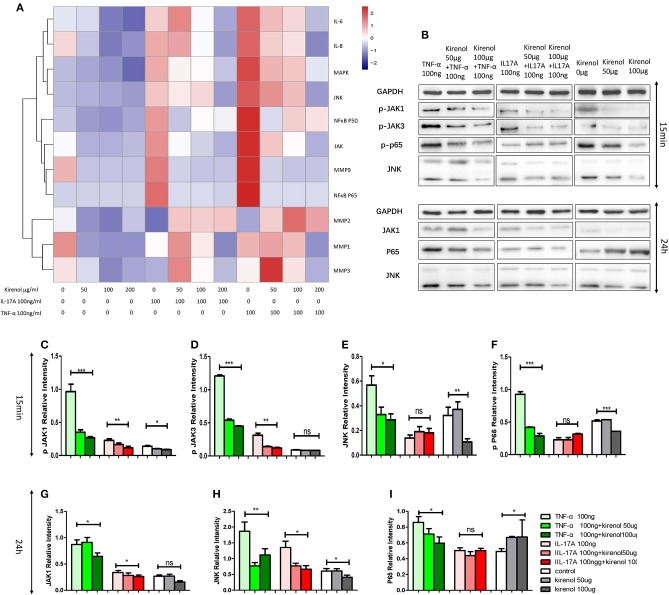
**(A)** IL-6, IL-8, MMP-1, 2, 3, 9, NFκB P65, P50, MAPK, and JAK expression in FLS stimulated with or without IL-17A and TNF-α. **(B)** Protein expression of JAK-STAT, NFκB, and MAPK pathway components in FLS stimulated with or without IL-17A and TNF-α. **(C)** Kirenol inhibited the phosphorylation JAK1 at 15 min. **(D)** Kirenol inhibited the phosphorylation JAK3 at 15 min. **(E)** Kirenol inhibited the phosphorylation JNK at 15 min. **(F)** Kirenol inhibited the phosphorylation NFκB-p65 at 15 min. **(G)** Kirenol inhibited JAK1 protein levels after 24 h. **(H)** Kirenol inhibited JNK protein levels after 24 h. **(I)** Kirenol inhibited NFκB-p65 protein levels after 24 h. *p < 0.05, **p < 0.01, ***p < 0.01 as assessed by the Kruskal-Wallis test.

RT-PCR was performed to assess the expression of IL-6, IL-8, MMP-1, 2, 3, 9, NFκBP65, P50, MAPK, and JAK in FLS cells treated with Kirenol. We found that expression of IL-6 and IL-8 were down-regulated by Kirenol in the presence or absence of IL-17A and TNF-α stimulation (Figure 2A). Similarly, MMP-9, NFκB, MAPK, and JAK were down-regulated by Kirenol, particularly following TNF-α stimulation. Kirenol had no apparent effect on MMP-1, 2, or 3 expression.

### Kirenol Down-Regulates JAK-STAT and NFκB but not MAPK Protein Levels in RA-FLS

To verify our RT-PCR results, Western blotting was next used to assess levels of key proteins in the MAPK, JAK-STAT, and NFκB pathways in these RA-FLS cells. We found that Kirenol affected the phosphorylation of JAK1 and JAK3 in the JAK-STAT pathways, as well as the phosphorylation of NFκB p65 in the first 15 min following TNF-α stimulation, after which no clear differences in protein phosphorylation were evident (Figures 2B–I).

### Kirenol Alters the Migration and Invasion of FLS

**Figure 3:**
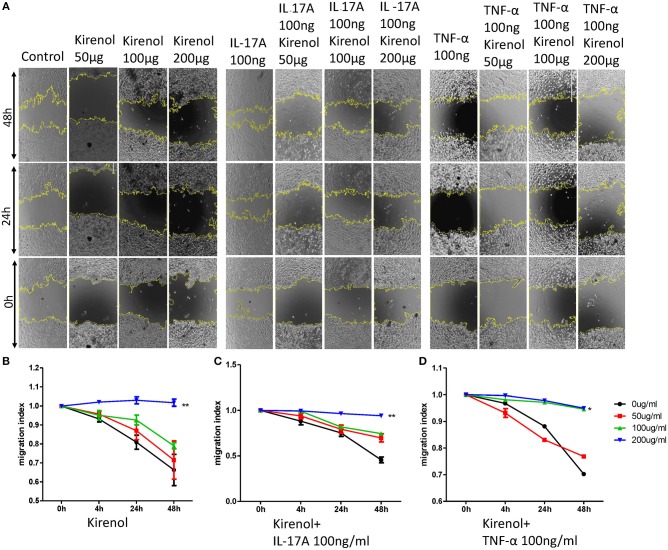
**(A)** Wound healing assay images; **(B)** Kirenol inhibited the migration of FLS in a dose-dependent manner, with significant differences only at 200 μg/ml; **(C)** Kirenol inhibited the migration of FLS stimulated with IL-17A (100 ng/ml) in a dose-dependent manner, with significant differences only at 200 μg/ml; **(D)** Kirenol inhibited the migration of FLS stimulated with TNF-α (100 ng/ml) in a dose-dependent manner, with significant differences only at 200 μg/ml; *p < 0.05, **p < 0.01, as assessed by Mann- Whitney *U*-test.

**Figure 4:**
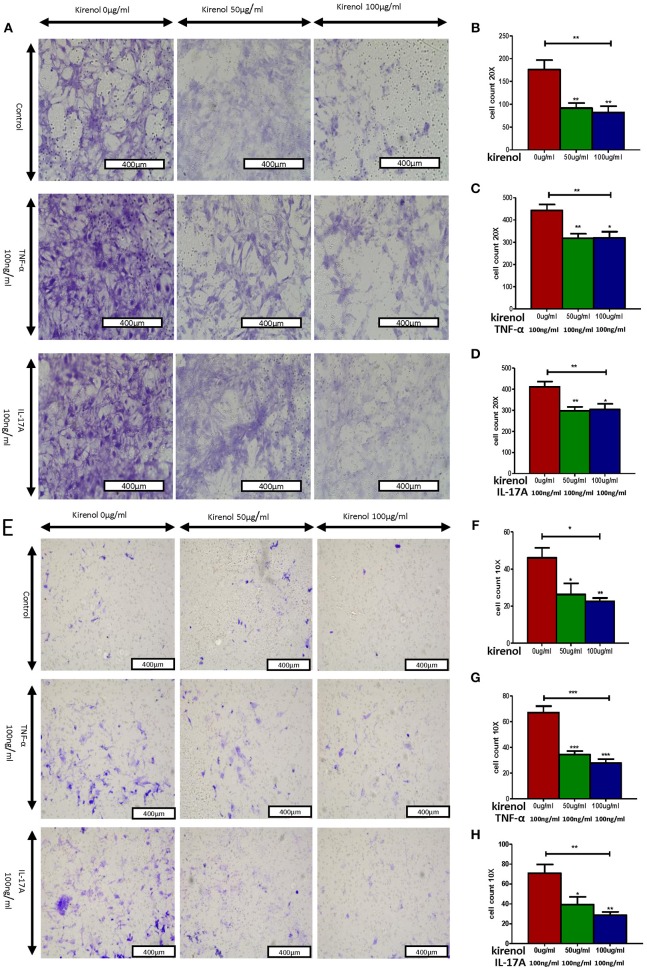
**(A)** Migration assay images; **(B)** Kirenol inhibited the migration of FLS; **(C)** Kirenol inhibited the migration of FLS stimulated with TNF-α (100 ng/ml); **(D)** Kirenol inhibited the migration of FLS stimulated with IL-17A (100 ng/ml); **(E)** Invasion assay images; **(F)** Kirenol inhibited the invasion of FLS; **(G)** Kirenol inhibited the invasion of FLS stimulated with TNF-α (100 ng/ml); **(H)** Kirenol inhibited the invasion of FLS stimulated with IL-17A (100 ng/ml); *p < 0.05, **p < 0.01, ***p < 0.01 as assessed by the Kruskal-Wallis test and the Mann-Whitney *U*-test.

We next assessed the effects of Kirenol on cellular migration and invasion, and found that it inhibited both activities even when cells were stimulated with IL-17 and TNF-α. In a wound healing assay, we found that the migratory ability of cells in the Kirenol-treated group was decreased compared with the control group (Figures 3A–D). Consistent with this, significantly fewer migrated cells were detected upon Kirenol treatment for the invasion assay (Figures 4A–H).

### Kirenol Alters Arthritic Progression *in vivo*

**Figure 5:**
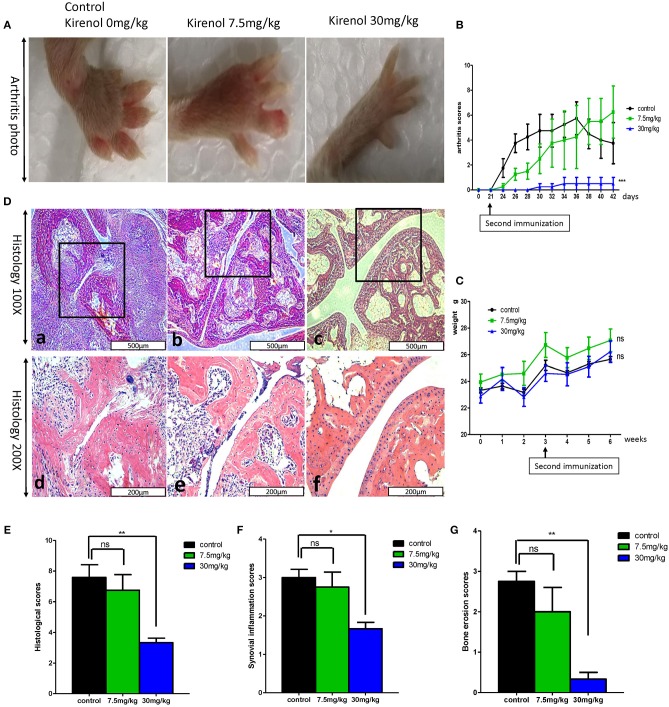
**(A)** Murine arthritis model (control(0 mg/Kg.d), 7.5 mg/Kg.d, and 30 mg/Kg.d Kirenol groups); **(B)** Arthritis scores for these three groups, with a significant difference between the control vs. 30 mg/Kg.d group; **(C)** Body weight did not differ significantly between groups. **(D)** a and d: Immunohistochemistry in the control group showed severe synovial hyperplasia and bone and cartilage destruction; b and e: Immunohistochemistry in the 7.5 mg/Kg.d group showed synovial hyperplasia and bone and cartilage destruction; c and f: Immunohistochemistry in the 30 mg/Kg.d group showed no serious synovial hyperplasia or bone/cartilage destruction; **(E)** Histological scores in the three groups, with a significant difference between the control group vs. the 30 mg/Kg.d group; **(F)** Synovial inflammation scores for the three groups, with a significant difference between the control group vs. the 30 mg/Kg.d group; **(G)** Bone erosion scores of three groups, with a significant difference between the control group vs. the 30 mg/Kg.d group; *p < 0.05, **p < 0.01, ***p < 0.01 as assessed by the Kruskal-Wallis test.

We next sought to extend our findings *in vivo*, in order to assess whether Kirenol was able to inhibit inflammation in a CIA mouse model of arthritis. We found that a low dose Kirenol (7.5 mg/kg) was able to delay the onset of arthritis, while a high dose (30 mg/kg) was able to reduce the incidence of arthritis (Figures 5A,B). Animals in the high dose group also exhibited reduced histological scores (Figures 5D–G), while body weight did not vary significantly at any tested dose (Figure 5C).

**Figure 6:**
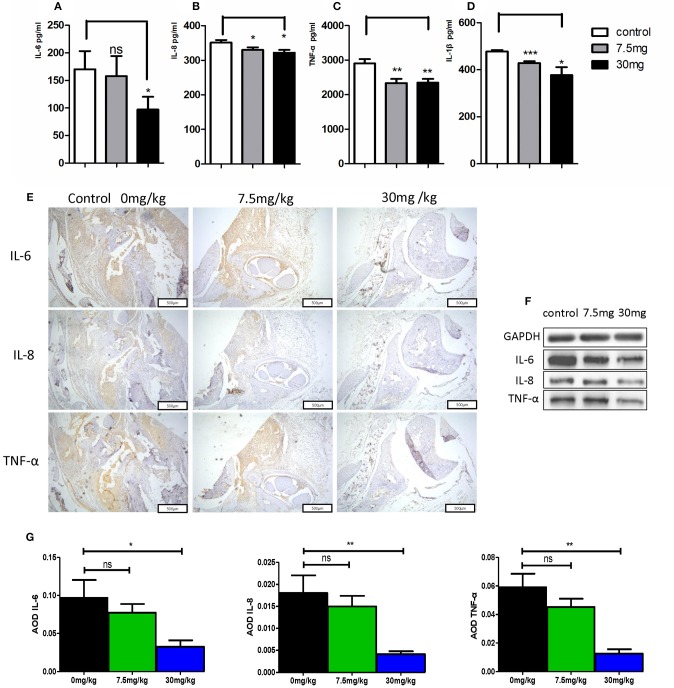
**(A)** IL-6 concentrations in the three groups, with a significant difference between the control group vs. the 30 mg/Kg.d group; **(B)** IL-8 concentrations in the three groups, with a significant difference between the control group vs. the 7.5 mg/Kg.d and 30 mg/Kg.d groups; **(C)** TNF-α concentrations in the three groups, with a significant difference between the control group vs. the 7.5 mg/Kg.d and 30 mg/Kg.d groups; **(D)** IL-1β concentration in the three groups, with a significant difference between the control group vs. the 7.5 mg/Kg.d and 30 mg/Kg.d groups; **(E)** Immunohistochemistry results indicated that Kirenol can inhibit the level sof IL-6, IL-8, and TNF-α in the synovium. **(F)** Western blotting indicated that Kirenol can inhibit the levels of IL-6, IL-8, and TNF-α in the synovium. **(G)** IL-6, IL-8, and TNF-α levels differed significantly between the 30 mg/kg group and the other experimental groups. *p < 0.05, **p < 0.01, ***p < 0.01 as assessed by the Kruskal-Wallis test and the Mann-Whitney *U*-test.

When murine serum was assessed via ELISA, we found that Kirenol was able to inhibit the production of TNF-α, IL-1β, IL-6, and IL-8 of in the serum (Figures 6A–D). Western blotting further confirmed that Kirenol was able to reduce levels of IL-6, IL-8, and TNF-α in the synovium, but only at the higher dose of 30 mg/kg (Figure 6F). Immunohistochemistry also confirmed that Kirenol can inhibit the levels of IL-6, IL-8, and TNF-α in the synovium, with differences only being significant at the 30 mg/kg dose (Figures 6E,G).

## Discussion

RA is characterized by the proliferation of synoviocytes in inflamed synovia, and by synoviocyte expression of inflammatory cytokines (15). FLS from RA patients exhibit extended hyperplasia, activation, and other aggressive behaviors such as abnormal migration and invasion (16, 17). As such, treatment strategies often focus on controlling the proliferation and inflammatory nature of these cells. Kirenol is a diterpenoid from Herba Siegesbeckia that has been used to treat RA for centuries. Kirenol has been suggested to exhibit anti-inflammatory and anti-rheumatic activities. Studies have found that Kirenol is effective in rat models of arthritis, but little is known about whether it can directly affect synovial cells. Therefore, in this study we designed a series of experiments to observe the effects of Kirenol on FLS both *in vitro* and *in vivo*.

*In vitro*, we found that Kirenol inhibited the proliferation and function of FLS in a dose-dependent manner. Interestingly, Kirenol exerted a more significant anti-proliferative and anti-inflammatory effect when these RA-FLS were first stimulated using TNF-α and IL-17. This confirms our previous findings indicating that Kirenol can dock the TNF-α (6). Furthermore, we found that only high-dose Kirenol (100–200 μg/ml) was able to affect FLS IL-6 secretion, even following TNF-α and IL-17A stimulation. By RT-PCR we further found that Kirenol can inhibit the expression of IL-6 at the mRNA level, indicating that high-dose Kirenol can readily alter IL-6 production by FLS. We were only able to detect inhibited IL-8 production at 30 min after Kirenol treatment in FLS, and such inhibition was absent in cells first treated with cytokines, although at the mRNA level this inhibition was evident. Even so, as these results are inconsistent, it is unclear whether Kirenol can substantially alter IL-8 production *in vitro*, particularly not in the context of a strong cytokine stimulus. We were not able to detect TNF-α or IL-1β ain FLS supernatants. We further found that RT-PCR that MMP-9 can be inhibited by Kirenol *in vitro*, whereas MMP-1,2, and 3 were not. We did not assess MMP protein levels in this study, and as such this is an important area of future research. We suspect that these proteins may be regulated by Kirenol, given previous work showing that this compound can inhibit MMP-2, 3, 9, and 13 expression in Hs68 human dermal fibroblasts (18).

To evaluate how IL-6 secretion and FLS function were affected by Kirenol, we next assessed the MAPK, JAK-STAT, and NFκB pathways in FLS cells, revealing that at early time points this compound inhibited the activation of JAK-STAT and NFκB but not MAPK signaling. Many studies have shown that TNF-α and IL-17A signaling through the NFκB pathway regulate the function of FLS in RA (19–21). We found that Kirenol inhibits the function of FLS in response to TNF-α and IL-17A, and so we hypothesized that Kirenol plays a negative role in controlling the activation of the NFκB pathway by blocking TNF-α and IL-17A signaling. Western blotting results were consistent with this hypothesis. Other researchers have also found that TNF-α signaling through JAK-STAT pathways can affect FLS responses (22), suggesting that Kirenol can inhibit responses to TNF signaling via multiple pathways, with similar inhibitory activities also likely in response to IL-6.

Some studies have found that RA-FLS migrate and invade cartilage and bone, leading to vascularization and tissue damage during RA progression (23). We found that Kirenol also inhibited the migration and invasion of FLS in a dose-dependent manner, even in response to TNF-α and IL-17A stimulation, which is significant as both cytokines can strongly promote cellular migration and invasion (24–27). We therefore believe Kirenol has a clear inhibitory effect on the migration and invasion of synoviocytes.

According to previous reports, Kirenol reduces the expression of cytokines in synovial and synovial fluid in a CIA rat model (28, 29). Histological evaluation in this study similarly revealed that Kirenol treatment effectively reduced joint inflammation, cartilage damage, and bone erosion, confirming that it helped to protect CIA mice. Our results are similar to those of other studies (5, 30). Moreover, we also found that Kirenol only achieved a protective effect at a dose of 30 mg/kg in this CIA mice model, with the lower dose only delaying the occurrence of arthritis and ultimately not affecting the disease outcome. No clear cytotoxicity or death was observed at any tested dose, indicating these treatment doses are safe. We further found that Kirenol significantly reduced the levels of TNF-α, IL-1β, and IL-6 in murine serum, confirming that this compound exhibits a therapeutic effect in CIA model mice.

Our *in vitro* and *in vivo* experiments have thus demonstrated that Kirenol has an excellent ability to inhibit synoviocyte functionality, suggesting that Kirenol has potential as a possible anti-rheumatic drug. This study is, however, limited by the fact that inflammation was only examined in the serum, joints, and synovium. Future studies will need to focus more broadly on how Kirenol affects the immune system *in vivo*.

## Conclusions

In this study, we found that Kirenol was able to strongly inhibit FLS proliferation, migration, and invasion, and to inhibit the release of pro-inflammatory IL-6 by FLS, even when these cells were activated with IL-17A and TNF-α. Kirenol is able to mediate this inhibitory activity in FLS via regulating various intracellular pathways. *In vivo* experiments further confirmed that Kirenol can inhibit bone erosion, synovial hyperplasia, and inflammation in the joints of arthritic mice in a dose dependent manner.

## Ethics Statement

The study is approved by Ethics Committee of ZhuJiang Hospital of Southern Medical University. The research design and methodology of this study were consistent with the requirements of the 2013 Helsinki Declaration. All study participants provided written informed consent.

## Author Contributions

JW, QL, LJ, HD, L-GJ, YQ, B-BL, and XZ performed experiments. JW and QL conceived the study and analyzed the results. JW and QY supervised the study and prepared the manuscript. All authors read and approved the final manuscript.

### Conflict of Interest Statement

The authors declare that the research was conducted in the absence of any commercial or financial relationships that could be construed as a potential conflict of interest.
